# Development of a Lateral Flow Immunoassay for the Rapid Diagnosis of Invasive Candidiasis

**DOI:** 10.3389/fmicb.2016.01451

**Published:** 2016-09-13

**Authors:** Zheng-Xin He, Lan-Chun Shi, Xiang-Yang Ran, Wei Li, Xian-Ling Wang, Fu-Kun Wang

**Affiliations:** ^1^Department of Clinical Laboratory, Bethune International Peace Hospital of PLAShijiazhuang, China; ^2^Department of Biochemistry, Bethune Medical NCO School of PLAShijiazhuang, China

**Keywords:** invasive candidiasis, lateral flow immunoassay, enolase, IgG, diagnosis

## Abstract

Early and accurate diagnosis of invasive candidiasis (IC) is very important. In this study, a lateral flow immunoassay (LFIA) was developed to detect antibody against *Candida albicans* enolase (Eno). Colloidal gold particle labeled mouse anti human IgG (1.0 mg/L) was used as the detector reagent. Recombinant enolase (rEno, 1.0 mg/L) and goat anti IgG (1.0 mg/L) were immobilized in test and control lines, respectively, of a nitrocellulose membrane, acting as the capture reagents. The LFIA was used to detect anti Eno in 38 sera from clinically proven IC patients, as well as in 50 healthy control subjects. Compared with an indirect ELISA designed as a reference test, the specificity and sensitivity of the LFIA were 98.2 and 84.8%, respectively. Excellent agreement between the results obtained by ELISA and the LFIA (κ = 0.851) was observed in this study. In addition, the agreement between the blood culture results and LFIA test is strong (κ = 0.658). The data presented in the study indicate that the LFIA test is a suitable tool for the serological surveillance of IC in the field or in poorly equipped laboratories.

## Introduction

Morbidity and mortality caused by invasive candidiasis (IC) including candidemia continues to increase worldwide in both immuno-compromised and immuno-competent patients (Pappas, 2006; Kullberg and Arendrup, 2015). A long-term intensive care unit (ICU) stay, abdominal surgery, acute necrotizing pancreatitis, hematologic malignant disease, and the use of broad-spectrum antibiotics are considered the leading factors for developing IC.

Early and accurate diagnosis for IC is very important for providing timely antifungal therapy in IC patients. Until recently, a blood culture has been considered the golden standard test for IC diagnosis (Clancy and Nguyen, 2013). A weakness of blood culture is its low sensitivity, which is estimated as low as approximately 50% considering *Candida* cells might be rapidly eliminated from blood circulation (Schell et al., 2012). Another notable shortcoming for blood cultures is that it is time consuming: the time to positivity can take as long as 8 days (Pfeiffer et al., 2011). Non-culture diagnostics were considered beneficial supplements for cases that are missed by blood culture. From one perspective, polymerase chain reaction (PCR) fulfills most of the criteria for clinical rapid IC diagnosis (Clancy and Nguyen, 2013). However, methodological standardization remains a primary concern for clinical study and use. Another approach for diagnosing IC is the detection of *Candida* cell components including β-D-glucan (BDG) and mannan. These tests are currently available in the commercial market and are usually used in combination with classical clinical, radiological, and microbiological findings for early diagnosis of invasive fungal infections (IFIs). However, false positive results of mannan or BDG-based detection tests have been described in many cases (Adam et al., 2004; Ostrosky-Zeichner et al., 2005; Zandijk et al., 2008).

Decade-long efforts have been made to establish a serologic diagnostic assay by detecting host antibodies against *Candida* components for rapidly diagnosing IC, but none are widespread in clinical use. Enolase (Eno), also known as phosphopyruvate hydratase, is a metalloenzyme responsible for catalyzing the conversion of 2-phosphoglycerate (2-PG) to phosphoenolpyruvate (PEP). For *Candida albicans*, this protein is present in both the cytoplasm and inner layers of the cell wall (Vialás et al., 2012). Previous works showed that this enzyme can elicit an antibody response in the infected host (Montagnoli et al., 2004; Pitarch et al., 2006). Among the molecular candidates for IC serodiagnosis, enolase is one of the most commonly studied, and it has a high diagnostic value. Several studies have demonstrated promising results for the diagnostic utility of detecting antibodies against Eno (Laín et al., 2007; Clancy et al., 2008; Li et al., 2013).

In this study, using recombinant Eno (rEno) of *C. albicans* (He et al., 2015), we developed a lateral flow immunoassay (LFIA) for the detecting human IgG antibodies against Eno. The test is rapid, easy to use and suitable for the serological diagnosis and surveillance of IC.

## Materials and methods

### Ethics statement

The human serum specimen study protocol was approved by the Ethics Committee of Bethune International Peace Hospital and complies with The Code of Ethics of the World Medical Association (Declaration of Helsinki) for experiments involving humans.

### Study population and serum specimens

All patients were admitted to Bethune International Peace Hospital, Shijiazhuang, China, from October 2011 to May 2015. Following the criteria of the European Organization for the Research and Treatment of Cancer/Mycoses Study Group [EORTC/MSG] (Ascioglu et al., 2002), 38 proven IC patients and 50 healthy subjects were enrolled in this study. For the proven IC patients, 38 strains of *Candida* pathogens were identified by blood culture, including 17 strains of *C. albicans*, 7 strains of *C. tropicalis*, 6 strains of *C. parapsilosis*, 4 strains of *C. glabrata*, 2 strains of *C. lusitaniae*, and 2 strains of *C. krusei*. To provide data on the assay specificity, the healthy subjects group has a similar age and sex distribution as the proven IC group.

For subjects with IC, the sample was collected within 72 h after positive blood culture results were obtained. This assured uniformity and that all subjects had active disease at the time of enrollment. All sera samples were stored at −80°C before analysis. Data, including the age, primary condition, and clinical stage, were obtained from the clinical records. Baseline characteristics of all subjects enrolled in this study are shown in Table 1.

**Table 1:** **Base-line characteristics of the 88 subjects included in the study**.

**Characteristic**	**Number (%) or mean ± S.D**.	
	**Invasive**	**Control**
	**candidiasis (*n* = 38)**	**subjects (*n* = 50)**
Age, mean years ±SD	58.6 ± 20.2	60.1 ± 18.6
**SEX**		
Male	25 (65.8)	32 (64.0)
Female	13 (34.2)	18 (36.0)
**PRIMARY CONDITION**		
Hematological malignancy	3 (7.9)	0
Solid tumor	6 (15.8)	0
Severe burn injuries	13 (34.2)	0
Respiratory dysfunction^a^	6 (15.8)	0
Gastrointestinal pathology^b^	7 (18.4)	0
Others^c^	3 (7.9)	0
**RISK FACTORS FOR IC**		
Broad spectrum antibiotics	22 (57.9)	0
Immunosuppressive therapy	11 (28.9)	0
Central venous catheters	7 (18.4)	0
Parenteral nutrition	8 (21.1)	0
Abdominal or thoracic surgery	9 (23.7)	0
Hematopoietic transplantation	1 (2.6)	0
Intensive care unit stay	9 (23.7)	0
Neutropenia^d^	4 (10.5)	0
Acute renal failure	2 (5.3)	0
**OUTCOME OF HOSPITAL STAY**		
Death	11 (28.9)	n.a.
Discharge	27 (71.1)	n.a.

a *Includes the following diseases: pneumonia, chronic obstructive pulmonary disease, and acute respiratory distress syndrome*.

b *Includes the following diseases: cholecystitis, angiocholitis, pancreatitis, and peritonitis*.

c *Includes the following diseases: acute renal insufficiency and diabetes mellitus*.

d *Defined as an absolute neutrophil count below 500 cells/mm^3^*.

*n.a., Not applicable*.

### Identification of *Candida* species

The collected blood samples were inoculated in a BacT/Alert 3D240 automated blood culture system (BioMerieux, France) for *Candida* growth detection. CHROMagar Candida medium was used to isolate and identify of *Candida* species from positive cultures. *Candida* species were further confirmed with a Vitek-2 system (BioMerieux, France).

### Generation of recombinant enolase

Recombinant enolase protein was generated and purified as described in our previous work (He et al., 2015).

### Preparation of detector and capture reagents

The detector reagent, 30-nm diameter colloidal gold particles labeled with mouse anti human IgG monoclony antibody (8 μg/ml), the gold solution pH was adjusted to 8.5. The test capture reagent, purified rEno protein, was diluted with 20 mM phosphate-buffered solution (PBS) (pH7.4) to 0, 0.5, 1.0, 1.5, and 2.0 mg/ml and stored at −20°C until required. Goat anti mouse IgG antibody was used as the control capture reagent and diluted to working concentrations of 0, 0.5, 1.0, 1.5, and 2.0 mg/ml with PBS (pH7.2). The diluted IgG solutions were stored at −80°C until later use.

### Preparation of the conjugate pad and immobilization of the capture reagents onto nitrocellulose membranes

The gold conjugated mouse anti human antibody solution was dispensed onto glass fiber paper (300 × 5 mm) at a speed of 10 μl per cm using an XYZ3050 Dispense Workstation (BioDot Shanghai, China), and the conjugate pad was dried under a vacuum. Different working concentrations of both the test and control capture reagents were dispensed onto nitrocellulose membrane strips (300 × 25 mm) at a speed of 0.80 ml solution per cm. After drying for 2 h at 37 C, the membrane strips were blocked by incubation in 20 mM PBS (pH 7.5) containing 2% (w/v) nonfat dried milk for 25 min and washed three times with PBS containing 0.1% (v/v) Tween-20 for 3 min at a time. The membrane was dried for 2 h at 37°C and stored at 4°C

### Preparation of the lateral flow immunoassay strip

**Figure 1:**
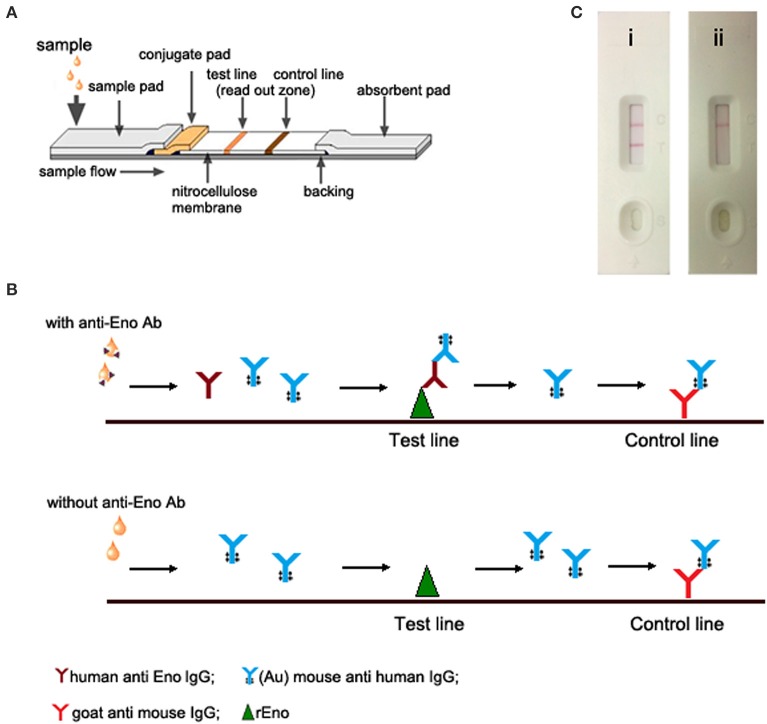
**Design of the lateral flow immunoassay strip. (A)** Schematic diagram. The conjugate pad was dispensed with gold conjugated mouse anti human antibody solution. At the test line and control line position, rEno and goat anti mouse IgG were immobilized, respectively. **(B)** The test principle of the lateral flow immunoassay strip. Human anti Eno IgG present in the sample react and bind to the gold conjugated mouse anti human IgG, then, it was captured by immobilized rEno, forming a positive result in the test line. Immobilized goat anti mouse IgG captures gold-conjugated mouse anti human IgG, forming the control line. **(C)** Interpretation of the results. i, positive (2 red bands at the readout zone) and ii, negative (only the control line area shows a red band).

The LFIA consisted of a sample pad, conjugate pad, immobilized nitrocellulose membrane, and absorbent pad. A schematic diagram of the LFIA is shown in Figure 1A. The prepared strips were stored dry at 4°C until required.

### Detection principle and test procedure

During the test, the sample solution was pipetted onto the sample pad. For a positive sample, the human anti Eno IgG reacted with the (Au)-mouse anti human IgG antibody conjugate to form a complex when the sample flows through the conjugate pad. Then, the complex was captured by rEno, forming a red band at the test line. Otherwise, no signal could be seen in the test line when a negative sample was used. Excess (Au)-mouse anti human IgG antibody conjugate binds goat anti mouse IgG in the control line, forming another red band (Figure 1B). Therefor, for the LFIA developed in this study, the appearance of two red bands in the read-out zone indicates a positive test and only one red band in the control line indicates a negative test (Figure 1C). In practical terms, the LFIA is laid on a flat bench and 100 μl of serum is added to the sample hole. The result is available in 15 min.

### Enzyme-linked immunosorbent assay (ELISA)

ELISA was performed as described in our previous work to detect the anti Eno IgG level in the sera of subjects enrolled in this research study (He et al., 2015). Briefly, rEno was adjusted to the concentration of 500 ng/mL in the bicarbonate buffer (pH9.6) and used to coat ELISA plates. After blocking with 3% BSA/PBS-T, 1:400 pre-diluted human sera was added to the plates and 1:10,000 diluted peroxidise conjugate goat anti human antibody was used as the secondary antibody. After the reaction was terminated with 2 N H_2_SO_4_, the microplates were read at a wavelength of 450 nm using an ELISA reader (VersaMax plate reader, Molecular Devices Co.).

### Specificity and sensitivity of lateral flow immunoassay strip and its agreement with reference methods

The strips were used to detect antibody in 38 IC patient serum samples and 50 control serum samples. Samples were tested by LFIA and ELISA in triplicate. The specificity, sensitivity, positive predictive value (PPV) and negative predictive value (NPV) of LFIA were calculated and compared with those of ELISA or blood culture results. The correlations between LFIA and ELISA or LFIA and blood culture were determined using κ statistical analysis (Viera and Garrett, 2005). Statistical analysis was performed using GraphPad Prism 5.0.

## Results

### Optimal concentrations of capture reagents for immobilization

Preliminary experiments established that 1.0 mg/mL goat anti mouse IgG was the optimal concentration for immobilization as the control capture reagent (data not shown). To optimize the concentration of the test capture reagent, rEno, at different concentrations of 0, 0.5, 1.0, 1.5, and 2.0 mg/ml was immobilized and used to test the sera from proven IC patients and control subjects. The corresponding test sensitivity, specificity and Youden index were calculated and are summarized in Table 2. The Youden test indicates the discriminatory accuracy of a diagnostic assay and offers an optimal cut-point value: we defined the optimal concentration of rEno as 1.0 mg/ml in this study.

**Table 2:** **Performances on sera using different test capture reagent (rEno) concentration**.

**Concentration**	**Sensitivity (%)**	**Specificity (%)**	Youden index^*^
0	0	100	0
0.5	29.0	100	0.29
1.0	71.1	95.0	0.66
1.5	76.3	84.0	0.60
2.0	84.2	74.0	0.58

* *Youden index was defined as J (t) = sensitivity + specificity −1*.

### LFIA test for blood culture positive and negative samples

A positive LFIA test result was found in 27 of 38 serum samples from proven IC patients, including 11 *C. albicans*, 5 *C. tropicalis*, 5 *C. parapsilosis*, 3 *C. glabrata*, 2 *C. lusitaniae*, and 1 *C. Krusei* infected sample(s). For the 50 control subjects without *Candida* infection, the LFIA test results were all negative, except two serum samples were determined as weakly positive. As a result, the sensitivity, specificity, PPV, and NPV of the LFIA diagnostic test based on detecting antibody against *Candida* enolase were 71.1, 96.0, 93.1, and 81.4%, respectively.

### Detection of Eno antibody by ELISA

**Figure 2:**
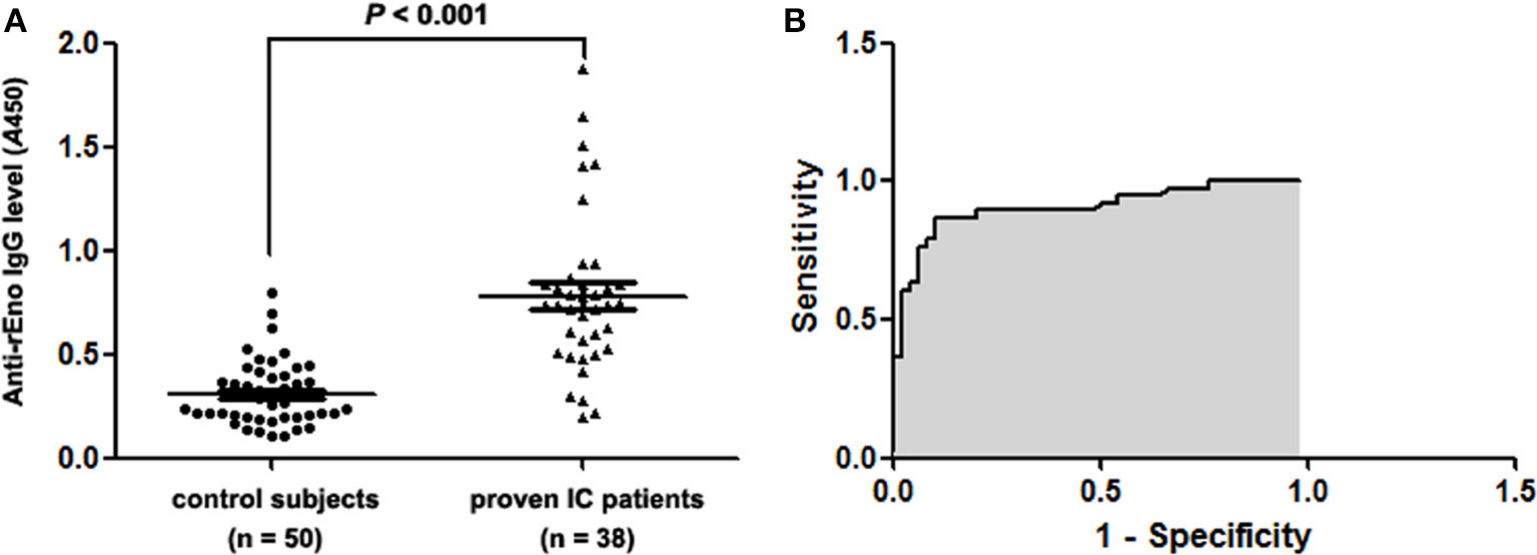
**ELISA test results for the study subjects**. **(A)** Antibody levels in the study subjects. The anti Eno levels for the sera from IC patients were significantly higher than those in control individuals (*P* < 0.001). **(B)** The receiver operating curve (ROC) was used to identify the anti Eno positive and negative subjects. The area under the curve (AUC) was 0.907 with a cutoff value of 0.476.

Figure 2A indicates the results obtained with the serum samples drawn from 38 IC patients and 50 control subjects who were tested by ELISA for detecting Eno IgG antibodies. The median of the antibody absorbance for sera from IC patients (median 0.734; interquartile range, 0.521–0.843) was significantly higher than that from control individuals (median 0.295; interquartile range, 0.203–0.388; *P* < 0.001).

To evaluate the predictive ability of the enolase antibody to the risk IC, we assessed the area under the curve (AUC) using the receiver operating characteristic curve (ROC) (Mandrekar, 2010). The sensitivity and specificity were calculated based on the ROC curve. The ROC curve for the enolase antibody in IC diagnosis is shown in Figure 2B and the AUC is 0.907 with a 95% confidence interval of 0.840–0.974. Considering a cutoff value of 0.476, the sensitivity, specificity, PPV and NPV of the ELISA diagnostic test were 86.8, 90.0, 86.8, and 90.0%, respectively.

### Agreement between the lateral flow immunoassay and reference methods

For the 88 clinical sera from IC patients and control subjects, 29 were antibody positive and 59 were negative according to LFIA, whereas 33 were positive and 55 were negative according to ELISA. Additionally, 28 were positive and 54 were negative in both tests. Therefore, the specificity and sensitivity of LFIA, compared with ELISA, were 98.2 and 84.8%, respectively. There was an excellent correlation (κ = 0.851) between the LFIA and ELISA.

The blood culture demonstrated 38 subjects were positive and 50 were negative (88 subjects in total), 27 subjects were determined positive and 48 were negative by both LFIA and blood culture tests. In addition, LFIA gave positive results for two blood culture negative samples and 11 negative results for blood culture positive subjects. A strong agreement (κ = 0.690) was observed between the blood culture and LFIA test results (Table 3).

**Table 3:** **Results of Eno antibody detection and clinical blood sample culture**.

		ELISA^*^					**Blood culture**		
		**Positive**	**Negative**	**Total**			**Positive**	**Negative**	**Total**
LFIA^*^	Positive	28	1	29	LFIA	Positive	27	2	29
	Negative	5	54	59		Negative	11	48	59
	Total	33	55	88		Total	38	50	88

* *ELISA, enzyme-linked immunosorbent assay; LFIA, lateral flow immunoassay*.

*LFIA compared to ELISA, κ = 0.851; LFIA compared to blood culture, κ = 0.690*.

## Discussion

IC is a serious cause of morbidity and mortality. Unfortunately, the laboratory technology for diagnosing IC cannot meet the current need for clinical applications (Clancy and Nguyen, 2013). To address the problem, the development of simple, rapid, sensitive and accurate diagnostic methods for IC has become an urgent focus.

As a rapid and simple detection method, LFIA has increasingly been used for detecting various human-associated infective pathogens (Ngom et al., 2010; Huang et al., 2016). In the present study, we developed a LFIA for diagnosing IC, which uses rEno protein to detect the corresponding antibody in the serum specimens of the IC patients. The rEno and a control antibody (goat anti mouse IgG) were immobilized on a membrane support as two distinct lines and were then combined with a sample pad, a conjugate pad that was impregnated with visualizing goad particles conjugated to monoclonal antibody of mouse anti human IgG and an absorbent pad to create the test strip.

Several studies have indicated that *C. albicans* enolase, a cell wall associated protein, is a promising candidate molecule for diagnosing IC, even in patients with neutropenia and immune defects (Laín et al., 2007; Clancy et al., 2008; Li et al., 2013). According to our previous work (He et al., 2015), enolase proved to be an immuno-dominant protein compared to other cell wall associated proteins, which makes enolase more applicable to serodiagnosis research. Using the rEno as the test capture reagent, we detect the antibody against enolase in the sera of proven IC patients and control subjects. When ELISA was used as a reference method, the LFIA test had good specificity (98.2%) and sensitivity (84.8%). There was excellent agreement between the results obtained by ELISA and the LFIA (κ = 0.851). In addition, the agreement between the results of the blood culture and the LFIA test (κ = 0.690) is also strong. These data suggest that there is an intense correlation between the enolase antibody levels and *Candida* species infection status, suggesting that LFIA would be valuable in IC surveillance.

For the ELISA test, the sensitivity, specificity, PPV and NPV were 86.8, 90.0, 86.8, and 90.0%, respectively. This result was similar to those seen in previous studies (Clancy et al., 2008; He et al., 2015). For the LFIA test, the corresponding values were 71.1, 96.0, 93.1, and 81.4%. The higher PPV (96 vs. 90%) by the LFIA is derived from the relatively higher false negative and lower false positive rates than ELISA. Maximizing the PPV is essential for reliably identifying patients who are likely to develop the disease, which should be considered when developing screening diagnostic methods. The LFIA test appears to be useful as a first-step assay for patients suspected of having IC, or as part of a diagnostic set in high-risk hosts. Clancy et al. (2008). reported patients infected with non-*C. albicans* species can be identified by responses against recombinant *C. albicans* antigens. In the present study, we detected IgG antibodies against recombinant *C. albicans* Eno in the sera from patients infected with non-*C. albicans* species. Because the sample size in our study is not sufficiently large to support effective statistical analysis, whether there is any relationship between false negative results given by LFIA and *Candida* species could be the subject of further research work.

Enolase is a highly conserved protein among different organisms (Van der Straeten et al., 1991; Sundstrom and Aliaga, 1992). When an immune response involving enolase was applied to the diagnosis, cross reactivity should be a source of concern. Laín et al. (2008) think that recombinant proteins could eliminate cross reactivity via posttranslational modifications. In a study conducted by Li et al. (2013), it is reported that sera from patients with bacteremia had no significant cross reactivity due to the lower antibody titers when diagnosing IC with rEno from *C. albicans*. Therefore, it can be expected to contribute low cross reactivity in the present study with patients with bacterial infections. It should be noted, however, that a shortcoming of the present study is that a large investigation with a sizeable sample size still should be conducted to confirm the aforementioned speculation.

Various methods have been developed for diagnosing IC, such as the ELISA protocol for antibody detection (Laín et al., 2007), β-D-Glucan detection (Ostrosky-Zeichner et al., 2005) and PCR (McMullan et al., 2008; Lucignano et al., 2011). However, these methods have common disadvantages of being time-consuming, and their uses are restricted to well-equipped laboratories. The LFIA reagent kit is rapid and easy to use. In this regard, the LFIA that we established is intended for use in the sero-surveillance of IC, especially in screening large numbers of blood samples in the field or poorly equipped laboratories. This would facilitate effective control of IC.

## Author contribution

ZH, FW conceived, coordinated and designed the study. ZH, LS contributed to the acquisition, analysis and interpretation of data, and drafted the manuscript. XR and WL performed the experiment and involved in drafting the article. XW participated in sample collection and data acquisition. All the authors have read and approved the final manuscript.

### Conflict of interest statement

The authors declare that the research was conducted in the absence of any commercial or financial relationships that could be construed as a potential conflict of interest.
